# An evaluation of the physiological uptake range of ^18^F-fluoro-2-deoxy-D-glucose in normal ovaries of seven dogs using positron emission tomography/computed tomography

**DOI:** 10.3389/fvets.2024.1343695

**Published:** 2024-02-02

**Authors:** Jinyoung Choi, Yeon Chae, Byeong-Teck Kang, Sungin Lee

**Affiliations:** ^1^Department of Veterinary Surgery, College of Veterinary Medicine, Chungbuk National University, Cheongju, Republic of Korea; ^2^Laboratory of Veterinary Internal Medicine, College of Veterinary Medicine, Chungbuk National University, Cheongju, Republic of Korea

**Keywords:** dog, ^18^ F-FDG, FDG uptake, ovary, PET/CT, physiological uptake range

## Abstract

**Introduction:**

This study evaluated the physiological uptake range of ^18^F-fluoro-2-deoxy-D-glucose (^18^F-FDG) in the normal ovaries of seven dogs using positron emission tomography/computed tomography (PET/CT).

**Materials and methods:**

The dogs were subjected to general anesthesia and were positioned in ventral recumbency for PET/CT scans. The dosage of ^18^F-FDG ranged from 0.14 to 0.17 mCi/kg and was administered intravenously followed by 0.9% NaCl flushing; PET/CT images of each dog were obtained precisely 60 min after the injection of ^18^F-FDG. The regions of interest were drawn manually, and standardized uptake values (SUV) were calculated to evaluate the ^18^F-FDG uptake in each ovary. The maximum and mean SUVs (SUV max and SUV mean) for all the ovaries of the dogs were then computed.

**Results:**

The range of SUV max and SUV mean of the normal ovaries of the dogs were 1.28–1.62 and 1.07–1.31 (mean ± standard deviation), respectively.

**Conclusion:**

This is the first study to investigate the normal ^18^F-FDG uptake baseline data of normal canine ovaries using PET/CT scans. These data will help clinicians in identifying malignant tumors before anatomical changes in the ovary through PET/CT scans.

## 1 Introduction

Positron emission tomography (PET) is a commonly used imaging technique in human medicine to help manage and evaluate the oncological status of organs (1–4). ^18^F-fluoro-2-deoxy-D-glucose (^18^F-FDG) is used as a radiotracer to help visualize the glucose metabolic rate in tissues (5, 6). This is because ^18^F-FDG is similar to glucose and migrates into the cells through glucose transporter proteins. However, unlike glucose, ^18^F-FDG becomes trapped within the cells because it is not converted into energy through glycolytic pathways (7–10). Generally, most malignant tumor cells have higher glucose metabolic activity than normal tissues (8, 10, 11). Consequently, PET has been utilized to detect malignancy and metastasis of tissues. PET scans operate by identifying malignant cells through the measurement of the uptake of 18F-FDG in a certain area, often before noticeable anatomical changes (12). Because of relatively low spatial resolution of PET, PET combined with computed tomography (PET/CT), which has a relatively lower spatial resolution, is an advanced imaging technique that helps provide superior anatomical information and maps the glucose metabolism of tissues through automatic image fusion (7, 13, 14).

PET/CT scans in human medicine have been widely utilized as a diagnostic tool across numerous fields. In cardiology, PET/CT scans serve as a clinically important diagnostic tool for characterizing the myocardial tissue viability of patients with coronary artery diseases (15). These scans can also be used to visualize non-tumoral bone and soft tissue disorders. This makes it a useful tool for the evaluation of orthopedic and rheumatologic diseases, allowing for more precise diagnosis and treatment planning (16). Furthermore, in neurologic medicine, PET/CT is a sophisticated technique that presents high spatial resolution and integrates functional data with morphological information. The information from the detailed images of PET/CT not only shows the precise location of abnormalities but also provides insights into the metabolic activity of the tissues. This information also helps in the diagnosis and management of CNS disorders (17). Additionally, there is an extensive research in human medicine on the degree of physiological ^18^F-FDG uptake of variable normal and tumorous tissues based on PET/CT (18, 19). Malignancy and functional statuses of the tissues have been identified by the measurement of standardized uptake values (SUVs) (11). Similar to the physiological SUVs in human tissues, the physiological SUVs of ^18^F-FDG in normal canine and feline tissues were reported previously (20, 21). However, no studies have been conducted on the physiological range of ^18^F-FDG SUVs in the reproductive tissues of dog ovaries.

This study aimed to investigate the ^18^F-FDG SUV uptake range of normal canine ovaries by examining the SUVs of seven dogs. This is the first article that demonstrated the physiological ^18^F-FDG uptake range of normal ovaries in dogs.

## 2 Materials and methods

### 2.1 Animal population

A total of seven intact female dogs weighing 9.9 ± 2.6 kg and aged 77 ± 38 months [mean ± standard deviation (SD)] were included in this study between October 2018 and July 2023 at the Veterinary Medical Center of Chungbuk National University. This study included four beagles, one Maltese, one Bichon Frise, and one mixed-breed dog. The dogs did not show any signs of metabolic diseases or other disorders. Before the injection of the radiopharmaceutical agent, ^18^F-FDG, each dog underwent a comprehensive assessment, including a physical examination, complete blood count, serum chemistry profile, abdominal ultrasound examination, and thoracic radiograph examination in the right lateral recumbent position, to help determine the size of the endotracheal tube required for inhalation anesthesia.

### 2.2 Anesthetic protocol

All dogs were fasted for 12 h before the induction of anesthesia and ^18^F-FDG injection. The blood glucose level was measured at 92.8 ± 8.9 mg/dL before the ^18^F-FDG injection. After the placement of peripheral intravenous catheter at the cephalic vein, the dogs were pre-medicated with midazolam (Midazolam, Bukwang Pharmaceutical Corporation, Seoul, Republic of Korea), and anesthesia was induced using propofol (Provive, Myungmoon Pharm, Seoul, Republic of Korea). After inducing anesthesia, endotracheal intubation was performed. General anesthesia was maintained using isoflurane (Terrell, Piramal Critical Care, Bethlehem, PA, United States), administered with 100% oxygen through inhalation anesthesia. Isoflurane within the anesthetic circuit was adjusted between 1.5 and 2.5% based on the dog’s vital signs (heart rate, respiratory rate, blood pressure, body temperature, oxygen saturation, and end-tidal carbon dioxide). All dogs in this study also received an intravenous infusion with lactated Ringer’s solution (Hartmann solution) at a rate of 10 mL/kg/h. A heating pad was also used to help dogs maintain their body temperature.

### 2.3 PET/CT acquisition

The PET/CT scan was performed under general anesthesia to evaluate the uptake of ^18^F-FDG in normal ovaries. The PET/CT technique used in this study for the PET scan was Discovery 72 STE (General Electric Medical Systems, Waukesha, WI, United States). PET/CT images from head to tail were obtained with an 8-slice helical CT scanner with 120 kVp, 150 mAs, 1.25 mm thickness interval, and 512 × 512 matrix of pixels. After the pre-contrast CT scan, post-contrast CT scan images were obtained 3 min after the intravenous injection of 880 mgI/kg iohexol contrast medium (Omnipaque, GE Healthcare, Marlborough, MA, United States). CT scan images of the ovaries were identified in transverse, coronal, and sagittal views. All dogs were administered ^18^F-FDG intravenously followed by 0.9% NaCl flushing for whole-body PET images. The dosage of ^18^F-FDG ranged from 0.14–0.17 mCi/kg and was administered intravenously slowly; 60 min after the injection of ^18^F-FDG, PET images of ovaries in dogs were obtained in five-bed positions and the ovaries revealed increased ^18^F-FDG uptake.

### 2.4 Image analysis

All images were analyzed using the commercial program OsiriX MD v11.0 (Pixmeo, Bernex, Switzerland) for image reconstruction and fusion of PET and CT images. The images were evaluated independently by three researchers (J. C., Y. C., and B.K.). The level of ^18^F-FDG uptake in the ovaries of all dogs was evaluated subjectively, and the regions of interest (ROIs) were drawn manually over the ovaries on the PET/CT scan images. The ROIs refer to specific areas or sections of the body that are the primary focus of the PET/CT scans. Consequently, three researchers individually identified the ovaries with ^18^F-FDG uptake in the fused PET/CT images and manually set the ROIs. In most ^18^F-FDG PET/CT studies, the standardized uptake value (SUV) is calculated to provide a quantitative measurement of ^18^F-FDG uptake within the ROIs. The SUV formula utilized was the average tissue concentration of ^18^F-FDG (MBq/ml)/injected dose (MBq)/body weight (g) for each ROI. In this study, maximum SUV and mean SUV within ROIs were computed to evaluate the ^18^F-FDG uptake in the ovaries.

### 2.5 Statistics analysis

The mean and standard deviations were assessed to interpret the data accurately and to provide insights into the central tendency and variability of the data, using IBM SPSS Statics version 22 (IBM, New York, United States). A 95% confidence interval (CI) is a range of values that can be 95% certain and contains the true mean of the SUV max and SUV mean of the dogs.

## 3 Results

**Figure 1:**
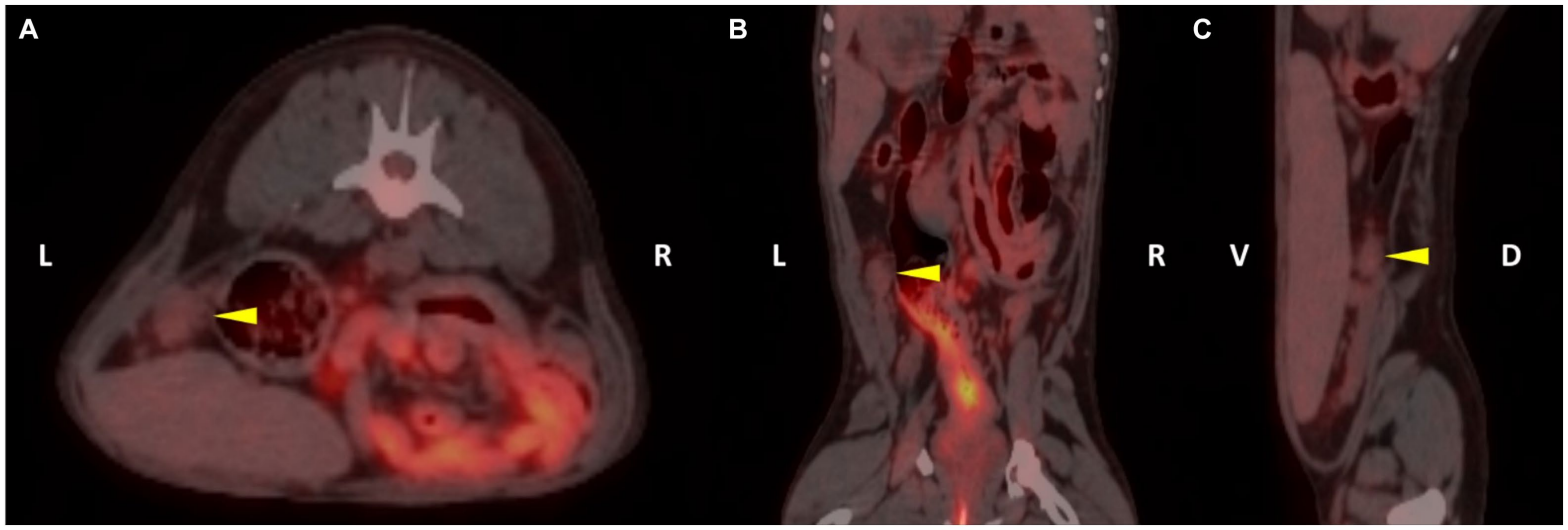
^18^F-fluoro-2-deoxy-D-glucose (^18^F-FDG) positron emission tomography/computed tomography (PET/CT) fusion images of patient 3. The left ovary of the patient was detected on the fusion images (yellow arrowhead). The regions of interest (ROI) were drawn in each plane to assess the ^18^F-FDG uptake of the left ovary (**A**, transverse plane; **B**, dorsal plane; **C**, sagittal plane).

The dogs showed stable vital signs such as no abnormalities in the heart rate, respiratory rate, blood pressure, or blood glucose during general anesthesia and acquisition of the PET/CT scan images. The range of post-scan glucose concentration in all dogs was 82.3 ± 8.2 mg/dL (mean ± SD). The dogs included were of small to medium size [body weight = 9.8 ± 3.6 kg (mean ± SD)] and of variable age [6.1 ± 6 years old (mean ± SD)] from four breeds. All dogs had normal ovaries based on CT and were fused with the PET after verifying the location and size of the ovaries to confirm the ^18^F-FDG uptake (Figure 1).

The characteristics and SUV max and mean values of ^18^F-FDG of the seven dogs are shown in Table 1. The ^18^F-FDG uptake of transverse, sagittal, and coronal image planes of bilateral ovaries was measured to quantitatively evaluate the amount of ^18^F-FDG. The mean and standard deviations with a 95% confidence interval for SUV maximum and mean within ROIs in the left, right, and bilateral ovaries are shown in Table 2.

**Table 1:** Characteristics and the maximum and mean SUVs of left and right ovaries in dogs.

Patients	Breed	Weight (kg)	Age (years)	Lt. ovary		Rt. Ovary	
				SUV max	SUV mean	SUV max	SUV mean
1	Beagle	10.4	3.2	1.532	1.378	1.441	1.134
2	Beagle	9.18	4.1	1.386	1.048	0.961	0.886
3	Beagle	13.48	4.4	1.615	1.191	1.926	1.373
4	Maltese	8.3	12.1	1.367	1.291	1.485	1.289
5	Beagle	8.46	5.8	1.48	1.321	1.367	1.092
6	Bichon Frise	8.4	3.7	1.637	1.311	1.592	1.323
7	Mixed breed	10.98	9.4	1.289	0.956	1.229	1.094

SUV, standardized uptake value. The regions of interest for each area were drawn manually over the ovaries and were analyzed by three researchers (J. C., Y. C., and B. K.).

**Table 2:** Results of maximum and mean standardized uptake values (SUVs) of ^18^F-FDG uptake measured using positron emission tomography for the left, right, and both ovaries of seven dogs.

	SUV max		SUV mean	
	Mean	95% CI	Mean	95% CI
Left ovaries	1.47	1.38–1.57	1.21	1.10–1.33
Right ovaries	1.43	1.20–1.65	1.17	1.05–1.30
Both ovaries	1.45	1.28–1.62	1.19	1.07–1.31

CI, confidence intervals.

The SUV max and mean values of the left ovary were 1.47 ± 0.13 and 1.21 ± 0.15 (mean ± SD), respectively. Similarly, the SUV max and mean values of the right ovary were 1.43 ± 0.30 and 1.17 ± 0.17 (mean ± SD), respectively. There was no significant difference between the SUV max and mean values of the left and right ovaries. Comprehensively, SUV max and mean values of the bilateral ovaries were 1.45 ± 0.22 and 1.19 ± 0.16 (mean ± SD), respectively. Overall, these SUV values could be considered the physiological range of ^18^F-FDG of normal canine ovaries.

## 4 Discussion

There are numerous studies in human medicine that evaluate the physiological ^18^F-FDG uptake in the reproductive organs based on PET/CT scans to distinguish between physiological processes and pathological conditions (22, 23). One PET/CT scan study on normal testis helped elucidate the normal physiological patterns of the ^18^F-FDG uptake and showed a correlation between the testicular ^18^F-FDG uptake, testicular size, and patient’s age (22). These baseline ranges can be used to differentiate the normal testicular function and the pathological change. In addition to studies investigating the uptake of ^18^F-FDG in normal testes and its correlation with testicular size, there has also been research on the relationship between testicular health and parameters such as total sperm count, sperm concentration, and motility (23). Research on ^18^F-FDG uptake in PET/CT scans has been extensive not only for male reproductive organs but also for female reproductive organs. In studies examining the ^18^F-FDG uptake in the human endometrium, the range of the ^18^F-FDG uptake in pre-menopausal women was established based on the four phases of the menstrual cycle including menstrual, ovulatory, proliferative, and secretory phases (24). Research has also been conducted on the physiological uptake of the ^18^F-FDG in the fallopian tubes, also known as uterine tubes. The uptake of ^18^F-FDG is more prominent in women during the mid-menstrual cycle. These findings suggest that the uptake of ^18^F-FDG in the fallopian tubes is influenced by ovarian hormones, particularly estrogen (25).

In this study, the physiological ^18^F-FDG uptake range on PET/CT scans of normal dog ovaries was established in seven dogs. In human medicine, in contrast to veterinary medicine, there are several reports on the ^18^F-FDG uptake of normal ovaries. One of the research studies has been conducted according to the phases of the menstrual cycle. The SUV of normal ovaries was 3.9 ± 0.7 in 26 pre-menopausal women during the follicular to luteal phase of the menstrual cycle. Furthermore, this study also identified that, in post-menopausal women, there was no observable ^18^F-FDG uptake in the ovaries and uterus (26). Compared to the SUVs of normal ovaries in the dogs identified in this study, the SUVs of human ovaries are higher. Based on these results, it can be inferred that there is a higher glucose metabolism in human ovaries compared to dog ovaries. Similarly, studies have found that the ^18^F-FDG uptake in the normal brains of dogs is measured to be lower than that in the normal human brains (21, 27). However, in the case of animals, a general anesthetic process is required for PET/CT scans, which is likely due to the glucose metabolic suppression during anesthesia (28).

The physiological range of the ^18^F-FDG uptake in the normal ovaries in dogs, as determined through PET/CT scans, can be effectively utilized in the assessment of patients with ovarian tumors. In PET/CT imaging, a higher ^18^F-FDG uptake indicates an increased metabolic activity as observed in many cancerous cells (7, 8). In patients with suspected ovarian tumors, PET/CT scans can help in differentiating between benign and malignant lesions by comparing their ^18^F-FDG uptake to the physiological range observed in normal ovaries before anatomical changes (2, 4). Tumors with ^18^F-FDG uptake significantly higher than the normal range are more likely to be malignant, while those within or close to the normal range may indicate benign tumors (10, 12). This method is particularly useful in the staging of ovarian cancer, planning treatment strategies, monitoring the effectiveness of therapy, and detecting recurrence (12). In human medicine, some studies have utilized ^18^F-FDG uptake in PET/CT imaging to assess the malignancy and metastasis of ovarian tumors (29–31). In addition to identifying the presence of tumors, a study conducted on ovarian tumor patients compared the SUV max to distinguish between borderline ovarian tumors and malignant ovarian tumors, which was found to be 2.9 ± 1.5 and 6.6 ± 2.9 (mean ± SD) (32). In veterinary medicine, ovarian remnant syndrome refers to a condition where the ovarian tissue remains in the body after ovariohysterectomy (33). There are several cases where these remnants can undergo tumorous changes, potentially leading to malignancy at an early stage (33, 34). Therefore, it has been established that, following a PET/CT scan, the determined ^18^F-FDG uptake in the ovaries can be utilized as a crucial diagnostic tool for identifying malignant ovarian lesions. The ability to quantify ^18^F-FDG uptake allows for a more precise assessment of the ovarian tissue.

There are some limitations of this study. First, this study was conducted with a small sample size for physiological uptake. Due to the small sample size, there is a potential for an increased margin of error and limited generalizability. Further studies using larger sample sizes could enhance the reliability of the study. Second, there are various factors that can influence the measurement of SUV calculation. Those factors include the size of ROIs, tissue or organ size, blood glucose concentration of the sample, and injection time of ^18^F-FDG (35). Third, there is a lack of information on the relationship between the ^18^F-FDG uptake and the physiological estrus cycle of dogs. In human medicine, some studies suggest that the menstrual status of pre-menopausal women can affect the physiological uptake of ^18^F-FDG on PET/CT scans (24, 26, 36). Furthermore, in post-menopausal women, normal ovaries demonstrate significantly less ^18^F-FDG uptake compared to those in pre-menopausal women (24). Additional research on ^18^F-FDG uptake throughout the canine estrous cycle will be helpful in the physiological ^18^F-FDG uptake range for normal ovaries. Finally, another limitation is that the ^18^F-FDG uptake on PET/CT can also be increased due to inflammatory or infected tissues and organs, which can lead to false-positive results (37).

In conclusion, this is the first study to investigate the physiological ^18^F-FDG uptake range of normal dog ovaries based on PET/CT scans. The ^18^F-FDG uptake range contributes to the baseline knowledge, which is necessary for the accurate interpretation of PET/CT scans in veterinary oncology, and provides a reference point to help distinguish physiological uptake from malignant transformation in canine ovaries. Therefore, malignant lesions of the ovary can thus be detected before structural changes, enabling the early diagnosis and assessment of patient prognosis.

## Data availability statement

The original contributions presented in the study are included in the article/supplementary material, further inquiries can be directed to the corresponding author/s.

## Ethics statement

The animal studies were approved by the Institutional Animal Care and Use Committee (CBNUA-2188-23-02). The studies were conducted in accordance with the local legislation and institutional requirements. Written informed consent was obtained from the owners for the participation of their animals in this study.

## Author contributions

JC: Conceptualization, Data curation, Formal analysis, Writing – original draft. YC: Conceptualization, Data curation, Formal analysis, Writing – review & editing. B-TK: Writing – review & editing. SL: Conceptualization, Formal analysis, Supervision, Writing – original draft.
